# Personalized medicine in the evaluation of Müllerian anomalies: the role of three-dimensional printing technology

**DOI:** 10.1016/j.xfre.2024.05.003

**Published:** 2024-05-16

**Authors:** Jessica Garcia de Paredes, Jordan Gosnell, Mili Thakur, Marcos Cordoba

**Affiliations:** a Department of Obstetrics, Gynecology and Women’s Health, Corewell Health, Grand Rapids, Michigan; b Department of Obstetrics, Gynecology and Reproductive Biology, College of Human Medicine, Michigan State University, Grand Rapids, Michigan; c Betz Advanced Visualization and Innovation Center, Corewell Health Helen DeVos Children’s Hospital, Grand Rapids, Michigan; d Betz Congenital Heart Center, Corewell Health Helen DeVos Children’s Hospital, Grand Rapids, Michigan; e Reproductive Genomics Program, The Fertility Center, Grand Rapids, Michigan; f Division of Maternal-Fetal Medicine, Department of Obstetrics, Gynecology and Women’s Health, Corewell Health Medical Group, Grand Rapids, Michigan

**Keywords:** 3D printing, 3D ultrasound, Müllerian anomalies, innovation

## Abstract

**Objective:**

To present the comprehensive methodology for generating personalized three-dimensional (3D) printed uterine models from 3D ultrasound (US) volumes in individuals diagnosed with Müllerian anomalies and discuss potential applications in the field of reproductive endocrinology and infertility.

**Design:**

Pilot study.

**Setting:**

Single large university-affiliated teaching hospital.

**Patient(s):**

Patients with the presence of a Müllerian anomaly between the ages of 18 and 45 years attending the maternal-fetal medicine as well as reproductive endocrinology and infertility outpatient offices from 2018 to 2023 were included in the study.

**Intervention(s):**

Subjects underwent 3D US transvaginal scanning for the collection of data. The 3D US volumes were acquired, edited, and exported from a US cart Voluson E10 system (GE Healthcare, Chicago, IL). High-definition virtual models were created and modified, making them suitable for printing using Materialise 3-Matic Medical (Materialise NV, Leuven, Belgium). The models were printed on a J5 MediJet 3D printer (Stratasys, Rehovot, Israel). Colors were set to mimic a realistic appearance, and shore values were set before printing.

**Main Outcome Measure(s):**

Successful creation and utilization of personalized 3D-printed uterine models for individuals with Müllerian anomalies.

**Results(s):**

Three-dimensional models were created for a uterus without anomalies, 2 variations of a partial septum, a unicornuate, and a didelphys uterus. Models were used as a tactile and customized tool for patient education, counseling, and medical student and resident teaching. This technique illustrates that the creation of personalized 3D-printed uterine models for utilization in the fields of reproductive endocrinology and infertility is feasible.

**Conclusion(s):**

We propose a novel use of individualized 3D-printed uterine models in the evaluation of individuals with Müllerian anomalies. These models may play a complementary role to standard imaging options in the assessment of these anomalies, with a special potential for application in highly complex or yet-to-be-determined types of anomalies.

Three-dimensional (3D) printing, which has been popularized in recent years with the advent of affordable desktop and personal 3D printers, originated 40 years ago as a form of rapid prototyping technology in the manufacturing industry (1). The increasing variety of technologies has gone hand in hand with a wide diversity of material types and physical properties (1). In medicine, the use of this transformative technology is increasing because of the need for improved visualization, personalized surgical planning, and the development of functional human organs and tissue (1, 2, 3, 4, 5, 6, 7, 8).

Prior studies have demonstrated several clinical advantages of 3D-printed applications, including improved medical outcomes, reduced surgical time, a better understanding of pathology, and reduced patient exposure to ionizing radiation (2). Three-dimensional printing has been used in multiple medical disciplines, giving rise to 3D-printed anatomical models, patient-specific guides, and personalized prosthetics (1). We have described previously that obstetrical 3D printing can optimize surgical planning, help with prenatal counseling, and deliver personalized care (3); however, the use of this technology remains limited in obstetrics and gynecology (1, 2, 3, 4, 5, 6, 7, 8, 9). There have been only a few reports of 3D printing in the field for antenatal counseling and surgical preparation of complex cases requiring a multidisciplinary team approach (5, 6, 7, 8).

The development of the female reproductive tract is a complex and dynamic process that involves a series of events, all of which are critical for proper tissue and organ differentiation (9). Deviations in these highly regulated signaling pathways translate into anatomical errors in the uterus, cervix, fallopian tubes, and vagina, all of which are incorporated into a spectrum of abnormalities known as Müllerian duct anomalies (MDA).

Müllerian duct anomalies represent rare developmental abnormalities of the female reproductive tract (9, 10). However, the prevalence of these anomalies is more common than previously thought. Earlier literature estimated that 1% of the female population, compared with 3% of females with poor reproductive outcomes, had a diagnosis of a Müllerian anomaly (11). Recent data has shown an increase in the known prevalence, with an estimate of 7% of anomalies involving the uterus and upper vagina, compared with as much as 25% in women who present with poor reproductive outcomes, namely infertility and multiple miscarriages (12, 13). Some explanations for the increase in prevalence may include the increase in the availability of more accurate imaging studies as well as a higher index of suspicion at the time of initial evaluation.

Despite the numerous existing classifications, the correct diagnosis, type, and management of these anomalies are often delayed or highly variable (10). The American Society of Reproductive Medicine outlines one important classification for MDA (10). Most but not all anomalies will fit into one of these categories with some overlap, creating a wide range of possible anomalies that are confusing and under constant study (10).

The complexity of the full spectrum of these anomalies poses a challenge for the training of residents and medical students, as well as during the counseling of patients about their anatomy. These challenges are faced not only by obstetrician-gynecologists but also by pediatricians, specialists in adolescent and emergency medicine, and general pediatric and urologic surgeons who may be involved in the care of these patients (10).

There is a need for patient advocacy and improved evaluation to prevent delays in diagnosis and treatment. To address this limitation, we have created personalized 3D-printed uterine models. We present a comprehensive methodology for generating these models on the basis of 3D ultrasound (US) volumes in individuals diagnosed with MDA and its potential applications.

## Material and methods

The most efficient method for 3D printing to date is to use US systems with the capability to directly export edited 3D US volumes in file formats commonly accepted by 3D printing software (1, 2, 4). Because of its ease of accessibility and safety, 3D US was the primary imaging modality utilized to create our models.

Inclusion criteria constituted individuals born with a uterus between the ages of 18 and 45 years with a Müllerian anomaly who attended the maternal-fetal medicine and reproductive endocrinology and infertility outpatient office from 2018 to 2023. This document uses the term patient to describe the included individual. This project was reviewed by the Corewell Health Institutional Review Board and was determined to be exempt because it did not meet the definition of human subject research (IRB No. 2021-534).

We selected uterine US volumes obtained during gynecological US examinations. Five patients were included in the study, and all patients underwent transvaginal ultrasound scanning. A RIC 5-9-D US transducer was utilized to collect high-quality two-dimensional images and 3D volumes. The US images were acquired using diagnostic US systems capable of 3D imaging and exporting volumes as image stacks in stereolithography (STL) format. The images were stored and extracted as Digital Imaging and Communication in Medicine data form and then transformed into an STL file format. The Magic Cut set was employed to clean and select the areas of interest for the precision of the 3D uterine model.

**Figure 1:**
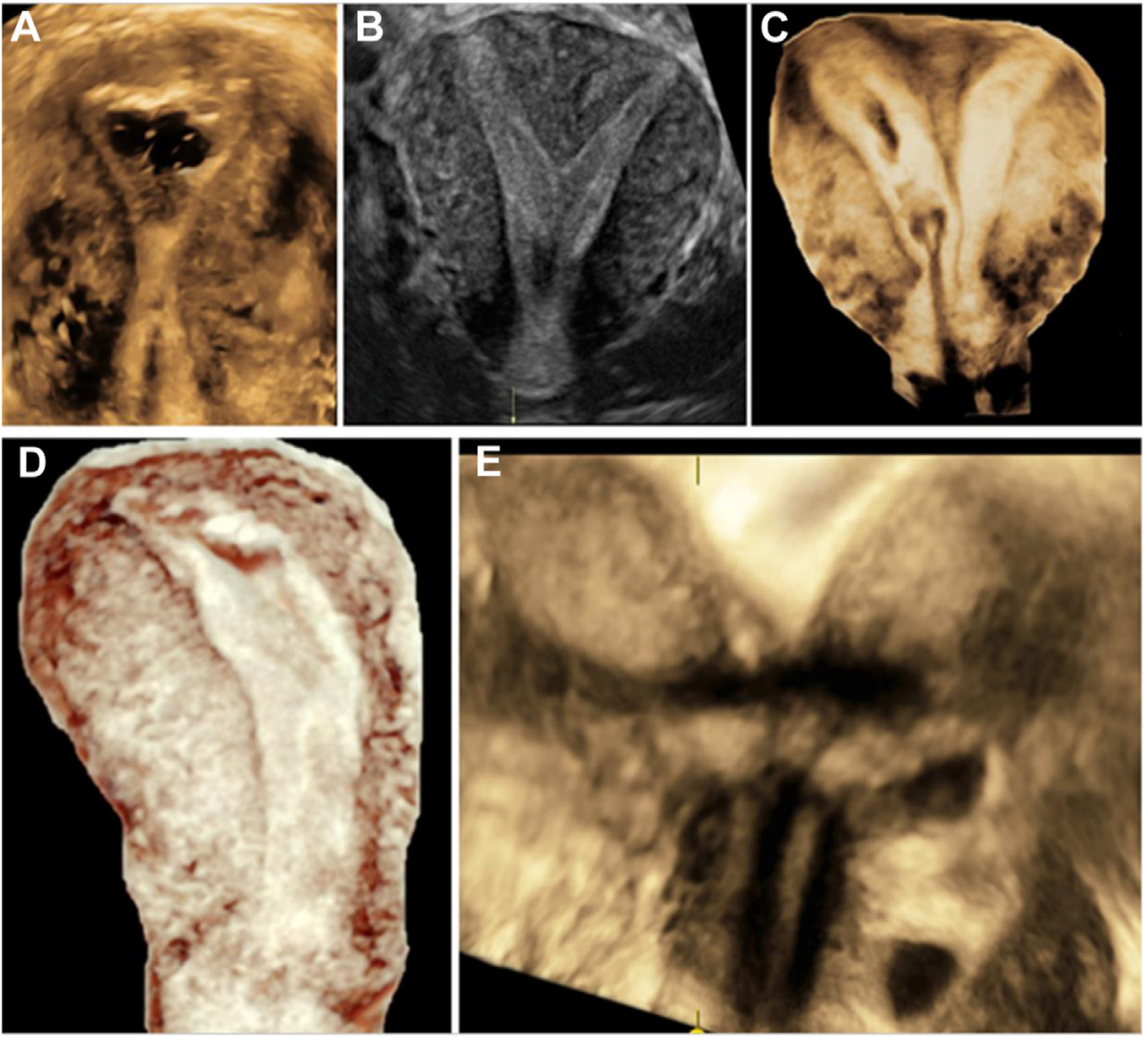
Three-dimensional ultrasound volumes for (**A**) the normal uterus, (**B, C**) two variations of a septum, (**D**) unicornuate, and (**E**) the didelphys uterus.

The 3D US volumes were acquired, edited, and exported from a US cart Voluson E10 system (GE Healthcare, Chicago, IL) for 3D ultrasonographic imaging and 3D printing data collection (Fig. 1).

**Figure 2:**
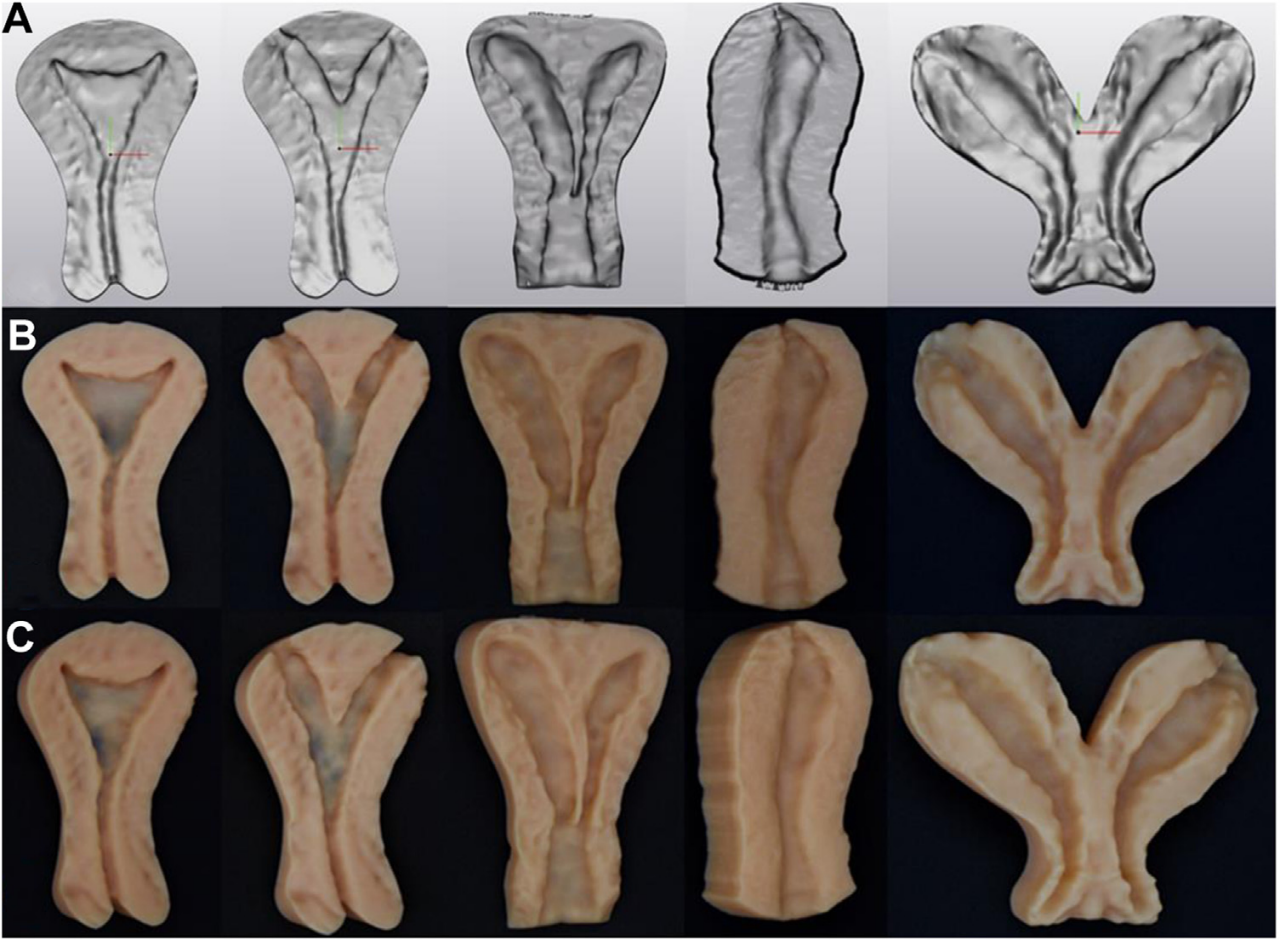
(**A**) Preprocessing high-resolution virtual models. (**B**) Front and (**C**) slightly tilted images of the postprocessing final version of the physical 3D printed uterine models, illustrating from far left to right—a normal uterus, two variations of a septate uterus, a unicornuate, and lastly, a didelphys uterus.

High-definition virtual models were created from the sourced US volumes (Fig. 2A). Generally, virtual imaging datasets cannot be directly 3D printed in their original state. Therefore, Materialise 3-Matic Medical (Materialise NV, Leuven, Belgium) was utilized to modify the source US imaging STL files to create a virtual model that could be viably printed as a cross-section bivalved physical anatomic model. We decided to proceed with this configuration to illustrate the internal anatomy unique to every personalized model, which is not able to be accomplished with an enclosed model and to give the students, providers, and patients the opportunity to “look inside” the uterus.

Primary operations to modify the virtual model included model wrapping, local smoothing, push and pull functions, hollowing (to conserve materials and allow for a more flexible model), and model labeling. The functions principally served to delineate the interior surface of the model, including the endometrial cavity, to best capture and illustrate the intricate topography of each anomaly and to be able to compare it with the anatomy of a uterus without any anomalies.

The models were printed on a J5 MediJet 3D printer (Stratasys, Rehovot, Israel). Colors to mimic a realistic appearance and shore values were set before printing in GrabCAD Print (Stratasys). The postprocessing included the removal of support material using an Objet WaterJet Cleaning System (Stratasys). The build volume of the J5 printer was able to accommodate and print all five models simultaneously.

## Results

Three-dimensional solid and slightly flexible uterine models were created for a uterus without anomalies, 2 variations of a partial septum, a unicornuate, and a didelphys uterus (Fig. 2B). The total print time was 14.5 hours. Personnel time dedicated to the creation of the models is estimated to be approximately 30 minutes of design time and 15 minutes of postprocessing time per model without including the image acquisition time of the 3D US volumes during transvaginal ultrasound scanning examination. The material cost per model ranged from $72.47 to $100.18, with a mean cost of 82.03 dollars per individual model.The models were utilized as a tactile and customized tool for patient education, counseling, and medical student and resident teaching. With the current and previously described techniques of 3D printing technology, we can demonstrate the possibility of the creation and potential uses of personalized 3D models in the fields of reproductive endocrinology and infertility.

## Discussion

Medical 3D printing is an innovative method for clinicians to visualize anatomic regions of interest by transforming anatomy traditionally viewed on a two-dimensional screen into a physically representative anatomic specimen. Multiple studies have demonstrated the clinical advantages of 3D-printed applications (1, 2, 3, 4, 5, 6, 7, 8). Although the entire potential of 3D printing in gynecology is yet to be fully realized, this technology holds promises for advancing surgical planning, personalized treatments, patient education, and medical training.

Prior modalities utilized in the creation of anatomical gynecological models include computed tomography (CT) scans and magnetic resonance imaging (14)*.* Most of the available data comparing software performance is limited to nongynecological settings and the utilization of CT scans or magnetic resonance imaging modalities. Materialise Medical software was selected because it is considered among the most used and best-known segmentation software, with prior studies showing statistically significant lower segmentation times and higher quality scores (15).

A recent study reviewing the utilization of 3D printing technology in gynecology reports the successful use of this software during the integration of 3D-printing noncoplanar templates during CT-guided ablative brachytherapy (16). However, there are no additional studies reporting the use of 3D US-based volumes in the creation of these anatomical models. Given the availability, reproducibility, safety profile, low cost, and decreased time burden to acquire the images, 3D US may represent a more cost-effective and overall safer alternative to the aforementioned modalities without compromising the quality of the models. The US would be also considered the study of choice during pregnancy.

In the past, neither US volumes in the proprietary format of US machines nor Digital Imaging and Communication in Medicine files could be used directly for 3D printing, and individual multisteps to manually convert the files to ready-to-print STL format needed to be undertaken, requiring a significant learning curve along the process (4). More recently, software versions permitting the automatic conversion of files are available, including the one utilized by our team (the VolusonTM E10 system), allowing the advantage of reducing the production time and increasing the ease of editing (4).

Still, streamlining the extraction of specific images from in-vivo 3D US examinations can be time-consuming. Sakas et al. (17) addressed this challenge by introducing an innovative clipping tool as an integral component of their ultrasound visualization system, solidifying its status as a foundational feature in contemporary commercial 3D US systems like GE Magicut. Through the selective removal of irrelevant regions, users can attain a clear view of typically obscured features, facilitating visualization and accurate assessment of anatomical structures, such as MDA. Once the area of interest has been meticulously refined through the clipping process, eliminating potential artifacts, the model is ready for postprocessing.

Three-dimensional printing utilizing 3D US volumes has been only recently described (4). We have modeled our workflow on the basis of prior methodology published by Tutschek et al. (4), describing the application of this technique in the 3D printing of live US volumes of abnormal and normal fetuses. Technical considerations were further modified during our prior experience in the utilization of patient-specific 3D printed models for antenatal patient counseling and surgical planning of a fetus with a large cervical mass in preparation for an exit procedure (3). Future studies are required and should be aimed at the validation of these models as well as the software utilized for their manipulation, with the goal of standardizing the process of image acquisition, manipulation, and creation of customized 3D uterine models in the setting of MDA.

### Additional applications: procedure planning

For the reproductive surgeon, presurgical planning, optimization of outcomes, and minimization of risks play a vital role in the surgical management of patients. With the use of this technology, surgeons at different stages of training can gain a better understanding of anomalous anatomy, visualize complex gynecological structures, assess potential challenges, and develop individualized surgical strategies with the opportunity to practice intricate surgical techniques in a simulated environment.

Reproductive specialists can use the 3D US technique along with 3D printing for surgical planning of complex pelvic and intrauterine surgeries such as MDA, severe intrauterine adhesions, and severe endometriosis. Besides, the 3D printed models constitute an additional tool available in the operating room to revisit at any time during the procedure and as a means for additional intraoperative education.

With increasing experience, 3D printing may be utilized to enhance surgical precision by designing and manufacturing customized surgical tools and instruments tailored to the specific needs of each patient in an affordable manner. Most importantly, as our project illustrates, these models can be printed in a timely and affordable fashion without delaying or financially burdening patient care, making it more accessible in the day-to-day surgical practice.

### Additional applications: medical training and patient counseling

Educational exposure to MDA is inconsistent given its rarity and low patient prevalence. Overall, reproductive endocrinology and infertility fellows graduate from their fellowship training with very little or no experience in performing complex hysteroscopic or laparoscopic procedures for MDA. To address this, 3D-printed models offer an alternative approach to constructing anatomically accurate models for any type of anomaly at any size and scale. Three-dimensional printing also offers the possibility of creating multiple copies of a certain pathology, standing the test of time, and being suitable for teaching across the globe. In addition, institutions can generate their own library of specialized and complex cases to use for future learners.

From the patient’s perspective, patient-specific 3D models enable them the opportunity to hold their pathology in their hands, providing the tools for a tangible representation of anatomical structures and pathological conditions. In turn, this allows patients to gain a better understanding of their pathology and proposed procedures, which may reduce decisional conflict and anxiety. This technology holds the potential to improve communication by empowering patients to actively participate in their treatment decisions and fostering a deeper sense of ownership over their health care journey. Plans include further studies documenting and comparing patient experiences using the models.

Although 3D printing has shown great potential in the field of gynecology, there are also several challenges that need to be addressed for its widespread adoption and successful implementation. Three-dimensional 3D US volume collection, as with any US-guided procedure, is user-dependent. Achieving the desired level of precision in reproducing complex gynecological structures can be challenging, and any inaccuracies can have significant implications for the generated models. This entails operator-related variations associated with the quality of the acquired images and US volumes, as well as limited allotted times for the acquisition of data.

At times, refractive shadowing at the edges or caused by echogenic structures between the transducer and the examined object may not permit sufficient detail to reconstruct all sides of an object (4). Because with “conventional” rendering from US volume data, volume acquisition from various angles and at different times may be used to alleviate this limitation (4). A full understanding of 3D US usually requires mastering volume acquisition techniques, volume manipulation programs, and personnel trained with the knowledge and ability to create these types of models. To make 3D printing accessible in gynecology, US units will require equipment, materials, maintenance, and expertise to enable users to export images in the format, allowing for the processing of the volumes to design high-definition virtual 3D models. In addition, the printers required to make high-quality 3D-printed physical models are expensive and not always readily available in every institution.

Multidisciplinary teams, including reproductive surgeons, radiologists, and 3D printing specialists, will require additional training to be able to incorporate 3D printing seamlessly into already existing clinical workflows. Because this technique makes its way into mainstream clinical practice, standardized protocols and validation processes for 3D printing in gynecology will become essential.

## Conclusion

We propose a novel use of individualized 3D-printed uterine models in the evaluation of individuals with Müllerian anomalies. These models may play a complementary role to standard imaging options in the evaluation of these anomalies, with a potential special application in highly complex or yet-to-be-determined types of anomalies.

The use of this technology has the possibility to translate into improved presurgical planning, patient counseling, satisfaction via individualized care, and the education of a multidisciplinary team. Further studies are needed to validate the quality and accuracy of the 3D-printed uterine models.

## CRediT Authorship Contribution Statement

**Jessica Garcia de Paredes:** Writing – review & editing, Writing – original draft, Methodology, Investigation, Conceptualization. **Jordan Gosnell:** Writing – review & editing, Writing – original draft, Software. **Mili Thakur:** Writing – review & editing, Writing – original draft, Investigation. **Marcos Cordoba:** Writing – review & editing, Writing – original draft, Visualization, Validation, Supervision, Software, Resources, Methodology, Investigation, Formal analysis, Data curation, Conceptualization.

## Declaration of Interests

J.G.d.P. has nothing to disclose. J.G. has nothing to disclose. M.T. has nothing to disclose. M.C. has nothing to disclose.
